# Enhancement of porcine intramuscular fat content by overexpression of the cytosolic form of phosphoenolpyruvate carboxykinase in skeletal muscle

**DOI:** 10.1038/srep43746

**Published:** 2017-03-02

**Authors:** Zijian Ren, Ying Wang, Yuanyuan Ren, Zhengwei Zhang, Weiwang Gu, Zhaoting Wu, Lingyi Chen, Lisha Mou, Rongfeng Li, Haiyuan Yang, Yifan Dai

**Affiliations:** 1 Jiangsu Key Laboratory of Xenotransplantation, Nanjing Medical University, 101 Longmian Avenue, Nanjing, 211166, People’s Republic of China; 2 Department of Biochemistry and Molecular Biology, School of Basic Medical Sciences, Tianjin Medical University, Heping District, Qixiangtai Road, Tianjin 300070, People’s Republic of China; 3 Huaian First Hospital Affiliated with Nanjing Medical University, Huai’an, People’s Republic of China; 4 Institute of Comparative Medicine and Center of Laboratory Animals, Southern Medical University, Guangzhou, People’s Republic of China; 5 State key laboratory of medicinal chemical biology, Key laboratory of bioactive materials, Ministry of education, Tianjin key laboratory of protein sciences and College of life sciences, Nankai University, Tianjin 300071, People’s Republic of China; 6 Shenzhen Xenotransplantation Medical Engineering Research and Development Center, Institute of Translational Medicine, Shenzhen Second People’s Hospital, First Affiliated Hospital of Shenzhen University, Shenzhen, Guangdong, 518035, China

## Abstract

Intramuscular fat (IMF) content has been generally recognized as a desirable trait in pork meat because of its positive effect on eating quality. An effective approach to enhance IMF content in pork is the generation of transgenic pigs. In this study, we used somatic cell nuclear transfer (SCNT) to generate cloned pigs exhibiting ectopic expression of phosphoenolpyruvate carboxykinase (PEPCK-C) driven by an α-skeletal-actin gene promoter, which was specifically expressed in skeletal muscle. Using qRT-PCR and Western blot analysis, we demonstrated that PEPCK-C was functionally expressed and had a significant effect on total fatty acid content in the skeletal muscle of the transgenic pigs, while the n-6/n-3 polyunsaturated fatty acid (PUFA) ratio showed no difference between transgenic and control pigs. Thus, genetically engineered PEPCK-C^mus^ pigs may be an effective solution for the production of IMF-enriched pork.

Pork is an important dietary source for humans, accounting for more than half of the world’s meat consumption. The competitive pork market is constantly changing based of consumer preferences and demands. Recently, the increasing consumer demands for higher eating quality of pork products has prompted changes in the pork industry^1^,^2^,^3^,^4^. Eating quality, a food property that encompasses taste, flavor, juiciness, and tenderness, can be affected by many physical and biochemical parameters. One parameter is intramuscular fat (IMF) content, which is generally believed to positively impact eating quality, although the data regarding this relationship appear to vary across studies^5^,^6^,^7^,^8^. Because IMF is generally associated with higher eating quality, the pork industry has a significant interest in augmenting the IMF content of pork through strategic feeding and genetic selection of pigs^9^,^10^,^11^,^12^,^13^,^14^. Strategic feeding has several disadvantages, including the need to develop specific feeding regimes for different pig breeds and the potential to change other aspects of meat quality besides eating quality^15^,^16^. Genetic selection is a promising approach, as IMF is a heritable trait in pigs, but this strategy is confounded by the existence of numerous quantitative trait loci (QTLs) that contribute to the IMF content^17^,^18^,^19^. Livestock genetic improvement programs could therefore benefit from a genetic engineering strategy to produce pigs with enhanced IMF levels^20^,^21^,^22^.

The cytosolic form of phosphoenolpyruvate carboxykinase (PEPCK-C) is a major rate-limiting enzyme in gluconeogenesis in the liver and kidney cortex and in glyceroneogenesis in the liver and white and brown adipose tissue^23^. The metabolic role of this enzyme in other mammalian tissues remains unclear. Hakimi *et al*.^24^ demonstrated that overexpression of the PEPCK-C gene in the skeletal muscle of mice increased triglyceride levels in skeletal muscle and decreased triglyceride levels in adipose tissue. This surprising metabolic outcome in mice prompted researchers to evaluate the developmental effects of the PEPCK-C transgene in livestock.

The objectives of this study were to use somatic cell nuclear transfer (SCNT) to generate transgenic pigs carrying a PEPCK-C transgene regulated by the α-skeletal-actin gene promoter and to determine the effect of the PEPCK-C gene on the IMF content of the transgenic pigs.

## Results

### Generation of PEPCK-C^mus^ transgenic pigs

**Figure 1:**
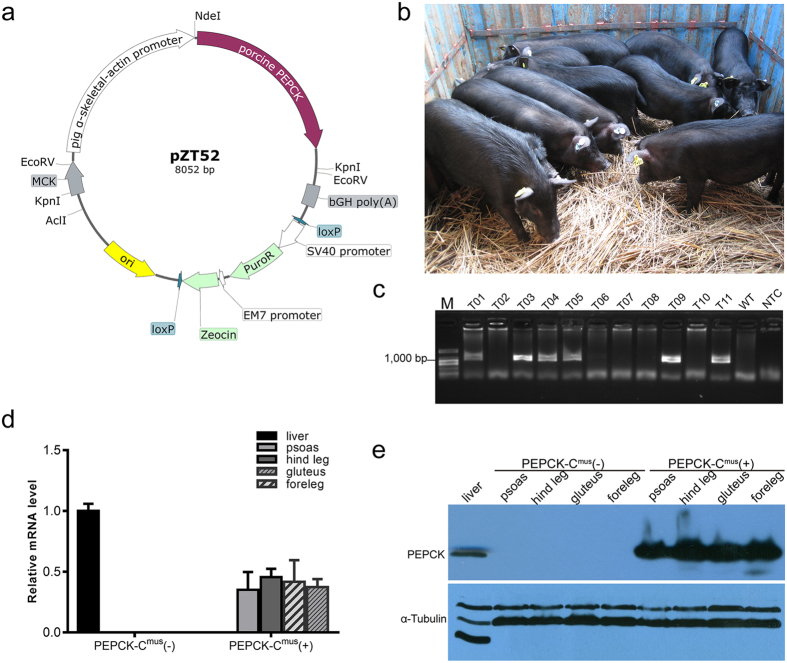
Generation and characterization of *PEPCK-C*^*mus*^ transgenic pigs. (**a**) Schematic diagram of the transgenic vector (pZT52). (**b**) Photograph of eleven cloned PEPCK-C^mus^ Tibetan mini pigs. (**c**) PCR analysis of genomic DNA from ear biopsies from the eleven cloned pigs. Six pigs (number T01, T03, T04, T05, T09 and T11) showed the expected 1,000 bp band. T01-T11, cloned pigs; M, marker; WT, wild type Tibetan mini pig as negative control; NTC, no template control. The full-length gel for this PCR analysis is presented in Supplementary Figure 1. (**d**) Real-time PCR analysis of PEPCK-C mRNA levels of skeletal muscle tissue from different anatomical locations (psoas, foreleg, hind leg, gluteus) of the transgenic and control pigs. PEPCK-C expression in wild type piglets’ liver tissue was selected as a positive control. Results were normalized to GAPDH. (**e**) Western blot analysis of PEPCK-C in skeletal muscle tissue from different anatomical locations (waist, foreleg, hind, gluteal) of the transgenic and control pigs. PEPCK-C expression in wild-type piglet liver tissue was selected as a positive control. α-Tubulin protein was used as an internal reference to demonstrate equal amounts of proteins were loaded. The full-length blots for this Western analysis are presented in Supplementary Figure 2 (PEPCK-C expression) and Supplementary Figure 3 (α-Tubulin expression).

Figure 1a shows a schematic of the PEPCK-C^mus^ transgenic vector used in this study. To construct this vector, a 1,869 bp cDNA sequence encoding the porcine cytosolic form of PEPCK, followed by the 3′ end of the bGH gene, was fused downstream of a 2 kb segment of the porcine α-skeletal-actin gene promoter. A SV40-puromycin expression cassette flanked by two *loxP* sites was linked to the construct as a selection marker. The total size of the plasmid was 8,052 bp.

Primary fetal fibroblasts derived from 35-day-old Tibetan miniature pig fetuses were transfected with the linearized PEPCK-C^mus^ plasmid and then screened by puromycin selection for approximately 10 days. Thirteen surviving single-cell clones were analyzed by PCR for integration of the porcine PEPCK-C gene. Nine clones harbored the expected 1,000 bp band. Colonies #5 and #26, which exhibited a higher quality and viability compared to the other colonies, were then selected to perform the SCNT procedure. In total, 640 reconstructed embryos were transferred into four surrogate pig recipients, and two recipients became pregnant. Only one surrogate pig receiving embryos from colony #26 delivered at full term, giving birth to eleven normal female piglets (designated T01 to T11, Fig. 1b) whose birth weights were comparable to those of the wild type piglets. The cloning efficiency was 1.7% (delivered piglets/transferred embryos). Among the eleven piglets, six (T01, T03, T04, T05, T09, T11) harbored the PEPCK-C transgene, as determined by PCR screening (Fig. 1c). These results indicate that six PEPCK-C^mus^ transgenic founder pigs were produced in this study.

### Expression of PEPCK-C in muscle tissue

Skeletal muscle tissues from different anatomical locations (psoas, foreleg, hind leg, and gluteal) were collected from two surviving founders (T03, and T05) for qRT-PCR analysis. Muscle samples from wild-type littermates (T02, T06, and T07) were used as controls. Due to the high levels of PEPCK-C in wild type piglet liver tissue, expression of PEPCK-C in the liver was selected as a positive control. PEPCK-C mRNA transcribed from the transgene was detected in all muscle tissue samples from PEPCK-C^mus^ pigs, while no transgene-transcribed PEPCK-C mRNA was observed in the control animals (Fig. 1d). These results indicate that the PEPCK-C gene was expressed efficiently under the control of the α-skeletal-actin gene promoter. Western blot analysis was performed with an antibody specific for PEPCK-C to assess the expression of the transgene in tissue samples derived from a transgenic pig (T05) and from a non-transgenic pig (T07). PEPCK-C was also detected in several tissues in both transgenic and wild type pigs, including heart, liver, and spleen tissues (Fig. 1e). However, consistent with the qRT-PCR results, PEPCK-C protein expression in skeletal muscle was only detected in the transgenic pig.

### Skeletal muscle of PEPCK-C^mus^ pigs exhibits increased lipid content

**Figure 2:**
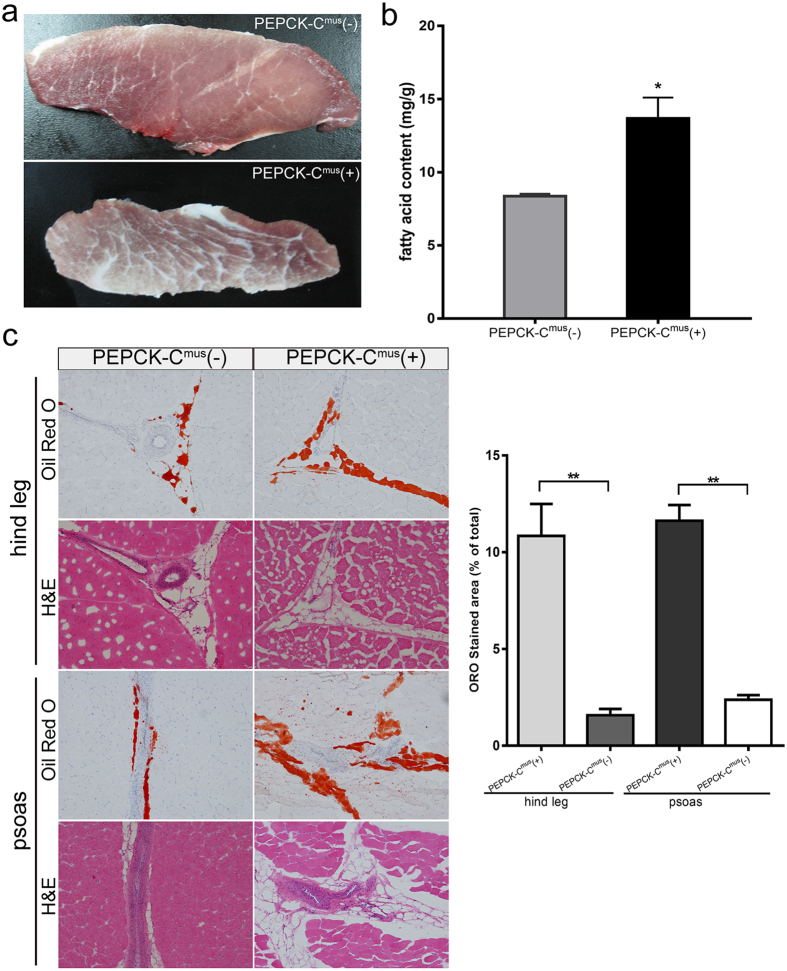
IMF was increased in PEPCK-C^mus^ transgenic pigs. (**a**) Representative images of the hind leg muscles from PEPCK-C transgenic and control pigs. (**b**) Left: Skeletal muscle tissue sections from transgenic and control pigs were stained with H&E (skeletal muscle) and Oil Red O (fat); Right: The Oil Red O stained area was quantified by ImageJ software. Data are shown as mean ± SEM (n = 3), **P < 0.01. (**c**) Total fatty acid contents of PEPCK-C^mus^(+) and PEPCK-C^mus^(−) pigs were determined by gas chromatography analysis. Values are presented as the mean ± SEM (n = 3) in two groups (*P < 0.05).

PEPCK-C overexpression produced a marbled pattern in the skeletal muscle of transgenic pigs (Fig. 2a). Hematoxylin/eosin staining and Oil Red O staining showed that the hind leg and psoas of PEPCK-C^mus^ pigs produced more lipids than that of control animals (Fig. 2b). The percentage of Oil Red O stained area occupied by IMF in the hind leg and psoas region was further quantified by ImageJ software. The results indicated that the Oil Red O stained area of the hind leg was approximately 6-fold higher in PEPCK-C^mus^ transgenic than in litter wild type pigs (PEPCK-C^mus^ pigs: 10.84 ± 1.65%, litter wild type: 1.58 ± 0.32%), and the Oil Red O stained area of the psoas was increased 5-fold in PEPCK-C^mus^ pigs compared with the litter wild type pigs (PEPCK-C^mus^ pigs: 11.63 ± 0.81%, litter wild type: 2.38 ± 0.24%) (Fig. 2b). Gas chromatography analysis was used to determine the total fatty acid content. Consistent with these histological results, in transgenic pigs, the average total fatty acids was13.68 mg/g, and the value was 8.36 mg/g in control piglets. Thus, there was significant increase of total fatty acids in transgenic skeletal muscle tissues (Fig. 2c). In addition to the total fatty acid content, we also determined the total amount of n-3 and n-6 polyunsaturated fatty acids (PUFAs) and the ratio of n-6/n-3 PUFAs in the meat samples because meat is a significant dietary source of PUFAs. PUFA composition of the hind legs of both control and transgenic pigs was measured by gas chromatography, and the data are shown in Table 1. The contents of n-6 PUFAs in transgenic pigs and litter wild type pigs were 22.17 ± 2.86% and 21.42 ± 2.40% of the total fatty acids, respectively, and n-3 PUFAs of transgenic pigs and wild-type pig comprised 1.67 ± 0.18% and 1.85 ± 0.23%, respectively, of the total fatty acids. However, the n-6/n-3 ratios showed no significant difference between transgenic and wild-type pigs.

## Discussion

In this study, we generated the first PEPCK-C^mus^ transgenic pigs by SCNT. Notably, the skeletal muscle of PEPCK-C^mus^ transgenic pigs had a higher triglyceride content compared to that of control animals, consistent with the skeletal muscle phenotype first observed in PEPCK-C^mus^ mice^24^. In the mouse study, this skeletal muscle phenotype was observed only in homozygous PEPCK-C^mus^ animals, whereas the founder mice expressed the transgene at varying levels. In contrast, in our study, an enhanced IMF phenotype was observed in PEPCK-C^mus^ transgenic founder pigs. This may be because the cloned transgenic piglets were derived from the same donor cell colony and their PEPCK-C expression was sufficient to exert PEPCK-C-induced metabolic effects in the skeletal muscle.

Since the transgenic pigs exhibited significantly increased IMF content in their skeletal muscle, we hypothesized that the eating quality of the pork derived from PEPCK-C^mus^ pigs would be increased, as IMF content is strongly correlated with improved pork quality^8^,^25^. However, a more detailed study is required to support this hypothesis. Although we are acutely aware of the safety measures that must be taken when introducing genetically-modified animals as a food source, safety concerns for PEPCK-C^mus^ transgenic pigs should be minimal, as the PEPCK -C gene used in this study originated from pigs, rather than from other species. Additionally, the selection marker in the PEPCK-C transgenic vector can be further excised by Cre-*loxP*-mediated recombination.

Previous studies have demonstrated that the longevity and reproductive capacity of PEPCK-C^mus^ mice were enhanced^24^. This study did not evaluate these parameters in our transgenic pigs due to the long-time course required for such experiments. However, these experiments are currently ongoing in our laboratory, and the results of these studies are anticipated to have significance to the pork industry, as the longevity and reproductive capacity of livestock are of great economic importance.

We also note that augmented IMF content in PEPCK-C^mus^ pigs may raise a health concern because fat is an undesirable component of meat for consumers. One solution to this issue may be crossing PEPCK-C^mus^ pigs with the mfat-1 transgenic pigs reported by Lai *et al*.^26^, which can produce meat products with improved eating quality and an optimal n-6/n-3 PUFA ratio. Additionally, our transgenic pigs showed no difference in the n-6/n-3 PUFA ratio from the control pigs. Taken together, genetically engineered PEPCK-C^mus^ pigs may be a valuable resource for the pork industry because of their potential to produce pork of high eating quality. Future studies should explore the application of the PEPCK-C transgene to the genetic improvement of other livestock species.

## Methods

### Ethics statement

This study was carried out in accordance with the guidelines approved by the Institutional Animal Care and Use Committee (IACUC) of the Nanjing Medical University, China. Tibetan mini pigs were housed in a large animal facility affiliated with Nanjing Medical University. All animals were fed chow diets purchased from Chia Tai (Jiangsu Huaiyin) Co., Ltd. twice a day. Standard pig husbandry procedures were applied to all animals.

### Construction of the transgenic expression vector

To construct the PEPCK-C expression vector, a 1.7 kb *EcoRV* fragment of pSNAP containing a *loxP*-Puro-Zeo-*loxP* cassette was inserted into a *PvuII*-digested pcDNA3.1/Myc-His A vector (Invitrogen, Carlsbad, USA) by blunt-end ligation. A 2 kb porcine α-skeletal-actin gene promoter was synthesized (Invitrogen, Carlsbad, USA) and subcloned into the *PvuI* and *NdeI* sites of the above plasmid. Subsequently, a 1,869 bp full-length cDNA sequence encoding porcine PEPCK-C was synthesized (Invitrogen, Carlsbad, USA) and inserted between the α-skeletal-actin gene promoter and the *loxP*-Puro-Zeo-*loxP* cassette after enzymatic digestion with *NdeI* and *KpnI*. Finally, a mouse MCK enhancer was amplified using the primers 5′-GATACGATCGACGGGCCAGATATACGCGTTAGAA-3′ and 5′-CCGGCGATCGGGCGGGCCATTTACCGTAAGTTAT-3′ and was then inserted into the *PvuI* site of the above vector. The completed vector, named pZT52, was then linearized with *AclI* and prepared for transfection into porcine fetal fibroblast cells.

### Generation of transgenic pigs by somatic cell nuclear transfer (SCNT)

Approximately 1 × 10^6^ Chinese Tibetan mini-pig fetal fibroblasts were transfected with 5 μg of the linearized transgene vector using the basic fibroblast nucleofection kit (Amaxa Biosystems/Lonza, Cologne, Germany) and nucleofection program U-023. Electroporated cells were replated in 10 cm dishes (Corning, Tewksbury, USA) at a density of 1 × 10^5^/10 mL after 48 h of recovery and selected with G418 (800 μg/ml) for 10 days. The antibiotic-resistant colonies were isolated to 24-well plates. Confluent cells were trypsinized with 100 μL of 0.25% trypsin and suspended in 350 μL of culture medium. Cells (200 μL) were plated in a 12-well plate, and the remaining cells were collected and lysed for PCR screening. The primers used for amplifying the PEPCK-C transgene were PEPCK-F (ACGGGCTCTACTGGAAGACA) and PEPCK-R (AGGCTGAAATTGCCAAGATG). PCR was performed for 35 cycles at 94 °C for 30 s, 60 °C for 30 s, and 72 °C for 30 s, and then held at 72 °C for 10 min. The transgene-positive clones were then pooled for later use in SCNT. SCNT was performed as previously described with minor modifications^27^. Briefly, to produce mature pig embryos for SCNT, ovaries from six-month old gilts were purchased from a local slaughterhouse. The oocytes were collected and cultured for 42–44 h for maturation. The *in vitro* matured oocytes were used as recipient cytoplasts after removing the cumulus cells and their nuclei. *In vitro* matured oocytes lacking cumulus cells and their nuclei and a single pig fibroblast with the PEPCK-C transgene were injected into the perivitelline space of the enucleated oocyte as the nucleus donor cell. After electrofusion of the membranes between the donor cell and recipient cytoplast, the reconstructed embryos were electrically activated and then cultured in embryo-development medium at 38.5 °C for 5 days until the blastocyst formed. Approximately 120 blastocysts were transplanted into the uterus of the surrogate pigs in estrus. The pregnancy status of the surrogates was monitored weekly by ultrasound starting one month after embryo transfer, and the cloned piglets were delivered by natural birth.

### Genotyping of transgenic pigs

Ear cells of the transgenic pigs were collected, and DNA was isolated from these cells by overnight lysis at 55 °C in a buffer containing 50 mM NaCl, 10 mM Tris-HCl, and 5 mg/mL proteinase K at pH 8.3. A PEPCK-C-specific primer pair was used to amplify a 1,000 bp fragment from the genomic DNA of the transgenic pigs (30 cycles: 94 °C for 30 s, 66 °C for 1 min, and 72 °C for 1 min). The PCR products were subjected to electrophoresis on a 1% agarose gel and stained with ethidium bromide.

### RNA extraction and quantitative RT-PCR

To quantify PEPCK expression levels in different tissues, total RNA was extracted from the tissues using the TRIzol RNA isolation system (Qiagen, Hilden, Germany). cDNA was synthesized from 2 μg of RNA using a High Capacity cDNA Reverse Transcription kit (TaKaRa, Dalian, China). cDNA was analyzed by real-time quantitative RT-PCR on an ABI Step One Plus real-time system (ABI, Foster City, USA) under the following thermal cycling conditions: 50 °C for 2 min for 1 cycle; 95 °C for 10 min for 1 cycle; and 95 °C for 15 s and 60 °C for 1 min for 40 cycles. All data were analyzed using GAPDH expression as a reference. Relative expression levels of PEPCK were calculated as previously described^28^.

### Western blot analysis

Western blots were performed with 20–50 μg of tissue extract. Proteins were separated by 8–12% SDS-PAGE and transferred to a polyvinylidene fluoride membrane (Millipore, Bedford, CA). The membranes were incubated for two hours at room temperature with an anti-PEPCK primary antibody (Cayman Chemical, Michigan, USA). Horseradish peroxidase-conjugated secondary antibody was detected with ECL substrate (Thermo Fisher, Wilmington, USA) in a bio-imaging system. Rabbit anti-PEPCK-C primary antibody (ab70359, Abcam, Cambridge, USA) and α-tubulin primary antibody (Sigma-Aldrich, St. Louis, USA) were used for protein detection.

### Histological analysis

Skeletal muscle was isolated from overnight-fasted PEPCK-C^mus^ and control pigs. The tissues were embedded in optimal cutting temperature (OCT) compound (Sakura, Torrance, USA). Consecutive frozen muscle sections of 10 μm thickness were cut and stained with Oil Red O to determine the content of the intramuscular fat. Briefly, frozen muscle sections were fixed in formalin and washed with tap water, followed by rinses with 60% isopropanol. Then, sections were stained with freshly prepared Oil Red O solution 15 min before nuclei staining with hematoxylin. Images were collected on an Olympus FSX100 microscope (Olympus, Tokyo, Japan). The stained area was quantified using ImageJ software (NIH, Bethesda, USA) and expressed as a percentage of the total image area. Another adjacent frozen muscle sections were used for H&E staining.

### Lipid extraction and fatty acid analysis

Lipids were extracted using a previously-reported method with some modifications^29^. Briefly, the muscle samples from different parts were homogenized in a mixture of methanol, chloroform, and water and then allowed to stand for 15 min. After the addition of more chloroform and water, the samples were vortexed and centrifuged. The lower phase was removed and dried under nitrogen and subsequently re-suspended in boron trifluoride methanol. The samples were heated at 90 °C for 30 min, extracted with pentane and water, resuspended in heptanes, and injected into a capillary column (SP2380 105 m × 0.53 mm × 0.20 μm, Supelco, Bellefonte, USA). Gas chromatography was performed using an Agilent 7890A gas chromatograph (Agilent Technologies, Santa Clara, USA). The components of the sample were identified by comparing their retention times with those of authentic standards (Sigma, St. Louis, USA). Fatty acid content is presented as percentages of total fatty acids in the muscle samples. The total amount of n-6 fatty acids was calculated from linoleic acid (LA, 18:2 n-6) and arachidonic acid (AA, 20:4 n-6). The total amount of n-3 fatty acids was calculated from a-linolenic acid (ALA, 18:3 n-3), eicosapentaenoic acid (EPA, 20:5 n-3), docosapentaenoic acid (DPA, 22:5 n-3), and docosahexaenoic acid (DHA, 22:6 n-3).

### Statistical analyses

Data from wild-type and PEPCK-C transgenic pigs were analyzed using unpaired Student’s t-test, and differences between the groups were considered statistically significant if *P* < 0.05.

## Additional Information

**How to cite this article**: Ren, Z. *et al*. Enhancement of porcine intramuscular fat content by overexpression of the cytosolic form of phosphoenolpyruvate carboxykinase in skeletal muscle. *Sci. Rep.* **7**, 43746; doi: 10.1038/srep43746 (2017).

**Publisher's note:** Springer Nature remains neutral with regard to jurisdictional claims in published maps and institutional affiliations.

## Supplementary Material

Supplementary Information

## Figures and Tables

**Table 1:** Polyunsaturated fatty acid composition of hind legs between control and transgenic pigs (% of total fatty acids).

	n-6 series					n-3 series				
	18:2	18:3	20:4	22:4	Total	18:3	20:5	22:5	22:6	Total
PEPCK(−)	16.89 ± 2.29	0.12 ± 0.01	4.42 ± 0.47	0.73 ± 0.09	22.17 ± 2.86	0.64 ± 0.05	0.17 ± 0.02	0.59 ± 0.08	0.27 ± 0.03	1.67 ± 0.18
PEPCK(+)	15.58 ± 1.55	0.12 ± 0.01	4.95 ± 0.74	0.78 ± 0.10	21.42 ± 2.40	0.55 ± 0.03	0.17 ± 0.02	0.67 ± 0.10	0.45 ± 0.08	1.85 ± 0.23

Values are shown as the mean ± SEM, n = 3.
