# Supplementary material for: Enhancement of porcine intramuscular fat content by overexpression of the cytosolic form of phosphoenolpyruvate carboxykinase in skeletal muscle
Source: Sci Rep. 2017 Mar 2;7:43746. doi: 10.1038/srep43746 (PMC5333075; doi:10.1038/srep43746)

Enhancement of porcine intramuscular fat content by overexpression of the cytosolic form of phosphoenolpyruvate carboxykinase in skeletal muscle

Zijian Ren[1*], Ying Wang[1*], Yuanyuan Ren[2*], Zhengwei Zhang[3], Weiwang Gu[4], Zhaoting Wu[5], Lingyi Chen[5], Lisha Mou[6], Rongfeng Li[1], Haiyuan Yang[1]✉ & Yifan Dai[1]✉

Supplementary Figure 1: The full-length gel of PEPCK transgenic piglets genotyping.

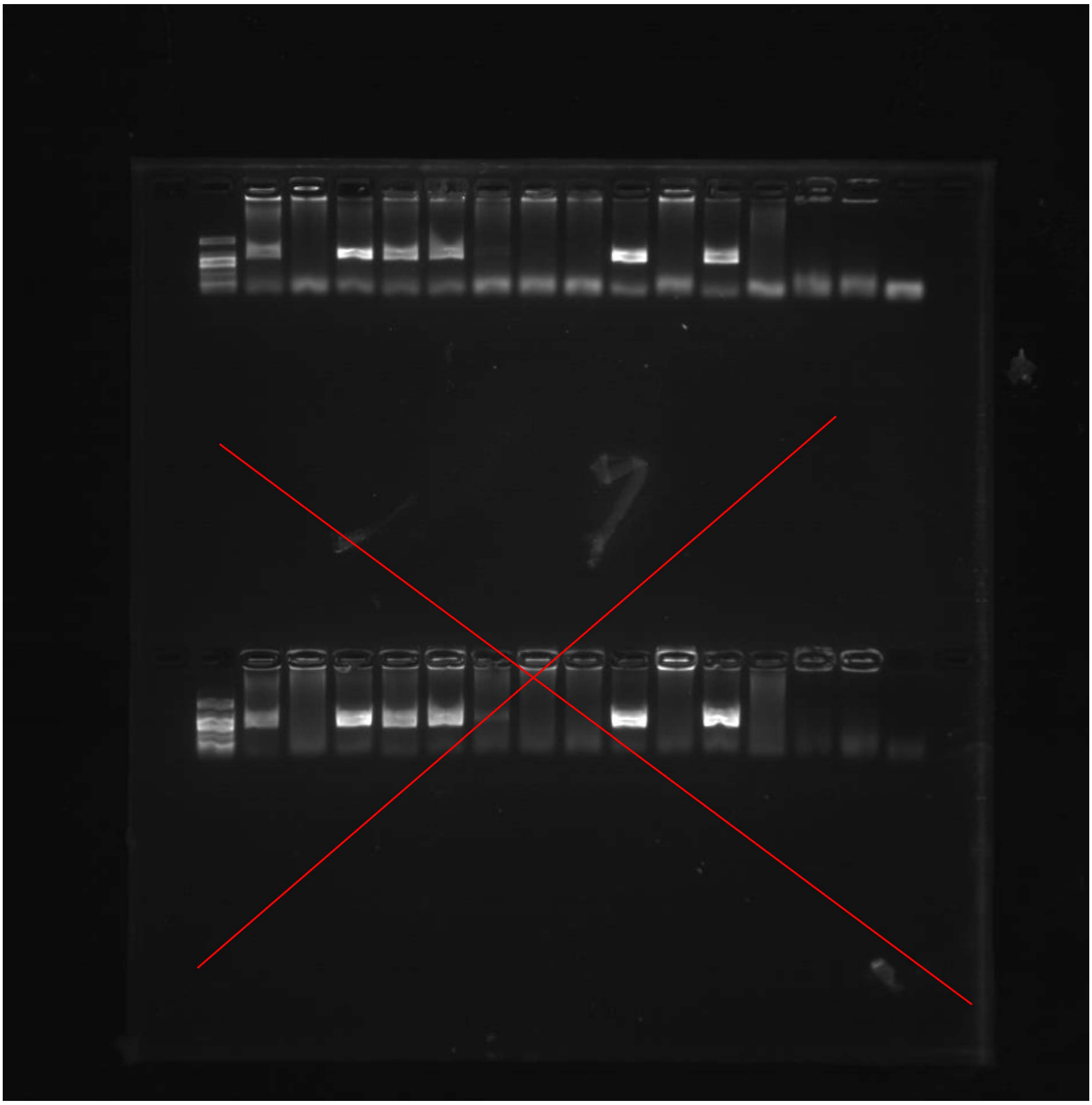

Supplementary Figure 2:The full-length blots for PEPCK Western analysis

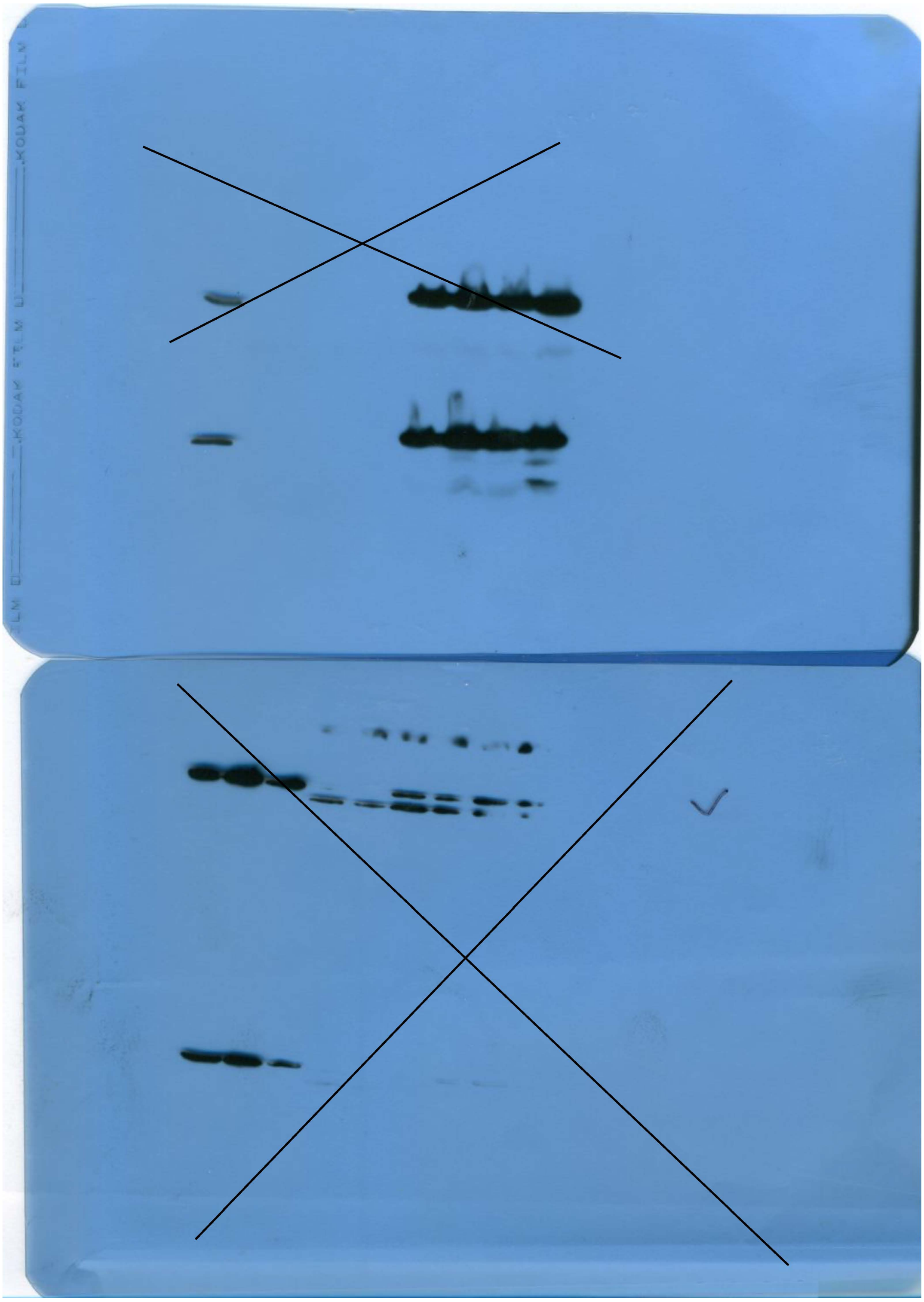

Supplementary Figure 3: The full-length blots for α-Tubulin Western analysis.

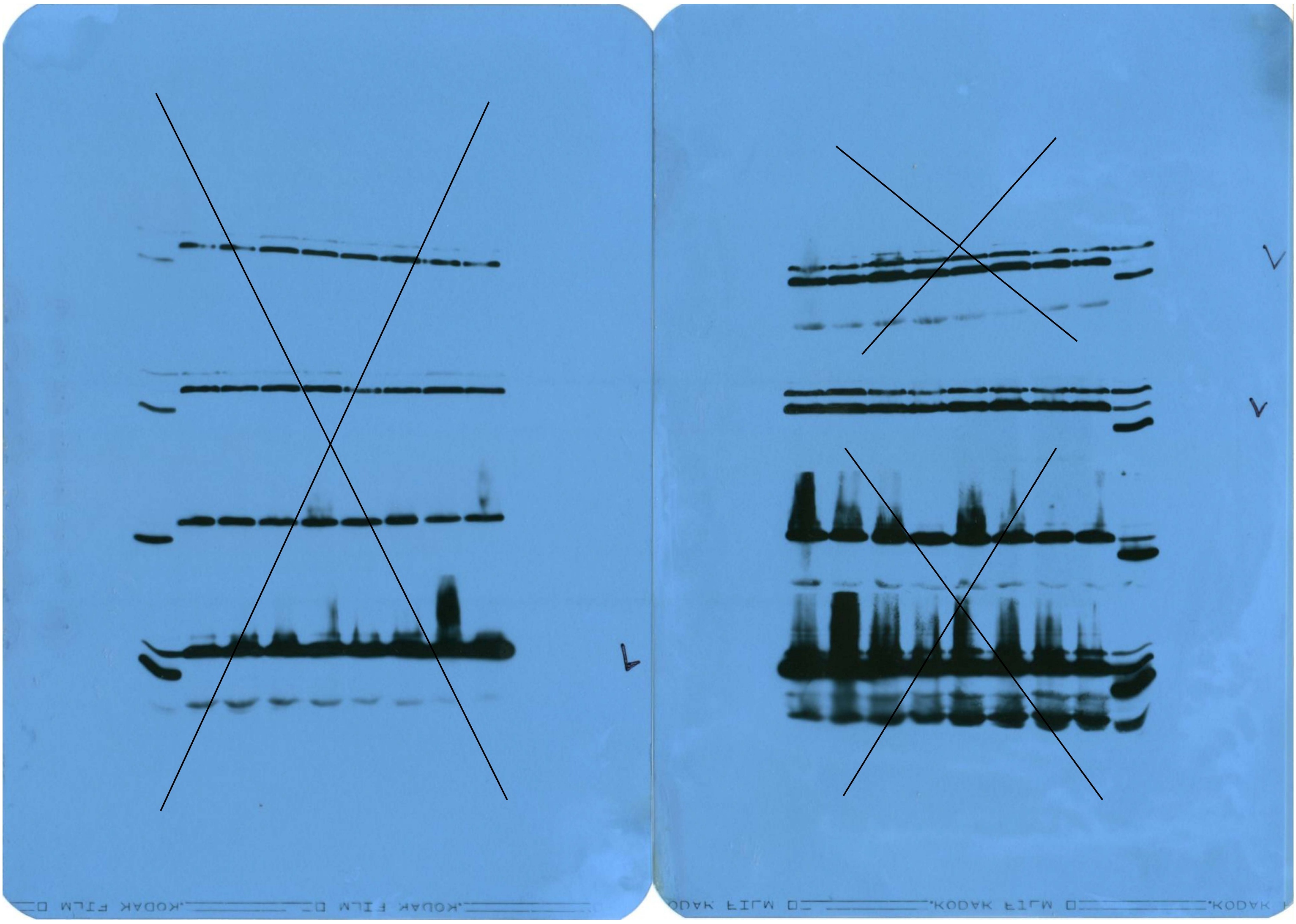

Supplement: Supplementary Information [file srep43746-s1.pdf]
